# Population health status of South Asian and African-Caribbean communities in the United Kingdom

**DOI:** 10.1186/1472-6963-12-101

**Published:** 2012-04-25

**Authors:** Melanie Calvert, Helen Duffy, Nick Freemantle, Russell Davis, Gregory YH Lip, Paramjit Gill

**Affiliations:** 1 MRC Midland Hub for Trials Methodology Research, University of Birmingham, Birmingham, UK; 2 School of Health and Population Science, University of Birmingham, Birmingham, UK; 3 University of Birmingham Centre for Cardiovascular Sciences, City Hospital, Birmingham B18 7QH, Birmingham, UK

**Keywords:** Health status, EQ-5D, South Asian, African-Caribbean

## Abstract

**Background:**

Population health status scores are routinely used to inform economic evaluation and evaluate the impact of disease and/or treatment on health. It is unclear whether the health status in black and minority ethnic groups are comparable to these population health status data. The aim of this study was to evaluate health-status in South Asian and African-Caribbean populations.

**Methods:**

Cross-sectional study recruiting participants aged ≥ 45 years (September 2006 to July 2009) from 20 primary care centres in Birmingham, United Kingdom.10,902 eligible subjects were invited, 5,408 participated (49.6%). 5,354 participants had complete data (49.1%) (3442 South Asian and 1912 African-Caribbean). Health status was assessed by interview using the EuroQoL EQ-5D.

**Results:**

The mean EQ-5D score in South Asian participants was 0.91 (standard deviation (SD) 0.18), median score 1 (interquartile range (IQR) 0.848 to 1) and in African-Caribbean participants the mean score was 0.92 (SD 0.18), median 1 (IQR 1 to 1). Compared with normative data from the UK general population, substantially fewer African-Caribbean and South Asian participants reported problems with mobility, usual activities, pain and anxiety when stratified by age resulting in higher average health status estimates than those from the UK population. Multivariable modelling showed that decreased health-related quality of life (HRQL) was associated with increased age, female gender and increased body mass index. A medical history of depression, stroke/transient ischemic attack, heart failure and arthritis were associated with substantial reductions in HRQL.

**Conclusions:**

The reported HRQL of these minority ethnic groups was substantially higher than anticipated compared to UK normative data. Participants with chronic disease experienced significant reductions in HRQL and should be a target for health intervention.

## Background

Black and minority ethnic groups (BMEGs) comprise 4.6 million (7.9%) of the UK population, the majority residing in deprived large metropolitan areas, as measured by the Index of Multiple Deprivation 2007 (IMD 2007) with greater Birmingham having the largest proportion of BMEGs outside London [1,2]. Birmingham has a population of nearly a million, 30% of whom are from the BMEGs. South Asians (i.e. Indian, Pakistani, Bangladeshi) and the Black African-Caribbean groups (i.e. from the Caribbean and Sub-Saharan Africa), as self defined using the 2001 Census Ethnic classifications, represent the largest minority ethnic groups in Birmingham and the UK [2,3].

In a clinical setting, multi-attribute health utility measures may be used to evaluate health status [4]. Such measures usefully allow the generation of a utility score (where 0 is a health state defined as equivalent to the state of death and 1 is full health, with negative scores indicating a health state worse than death). These scores can be used in combination with the time spent in a health state to generate Quality Adjusted Life Years and used as a measure of effectiveness in economic evaluation. Utility measures such as the EQ-5D, SF-6D, Health Utilities Index and Quality of Well-Being Scale may be used to evaluate health status in both the general population and in clinical trials to evaluate the effect of disease and response to treatment [5-8].

The health status of the UK population has been evaluated based on a stratified random sample (n = 3395) of the UK general population aged 18 or over using the EuroQoL EQ-5D questionnaire in 1993 [9,10]. The 'descriptive population norms' produced in this study have been used extensively to 'provide baseline values for monitoring variations in health' and to inform economic evaluation. The ethnicity of participants included in the UK population study was not described but given the 1991 census results minority ethnic groups are likely to comprise a small proportion of the sample (< 6%). In the 1991 census over 3 million people (5.5% of the population) identified themselves as belonging to one of the non-white ethnic groups. South Asians (Indian, Pakistani, and Bangladeshi) together formed 2.7% of the British population. The Black ethnic groups accounted for 1.6% of the population [2].

The aim of this study was to evaluate the HRQL of South Asian and African-Caribbean subjects who were enrolled in the Ethnic-Echocardiographic Heart of England Study (E-ECHOES) study [11].

## Methods

### Study population

The design and protocol of the E-ECHOES study including details of the sample size and analysis plan have been published [11]. The Walsall Local Research Ethics Committee reviewed and approved the protocol (05/Q2708/45). In brief, this was a cross-sectional population survey of a sample of South Asian (SA) South Asians (i.e. Indian, Pakistani, Bangladeshi) and the Black African-Caribbean groups (AC) (i.e. from the Caribbean and Sub-Saharan Africa), as self defined using the 2001 Census Ethnic classifications, male and female residents of Birmingham aged 45 years and over [11]. All SA and AC residents, including those born in the UK or immigrants, identified from 20 health centres, in inner city Birmingham, UK, were invited to participate. Multiple methods were used to identify subjects as ethnicity data is not routinely collected in primary care. Potential SA participants were identified using the Nam Pechan software based upon subject name and visual inspection by PSG [12]; and for AC subjects practice staff were consulted. Lists were reviewed by the general practitioner to ensure that only SA and AC subjects were included. Residents with dementia or terminal illness were excluded; however, no further selection criteria were applied in relation to medical history. Potential subjects were mailed an invitation letter and/or telephoned up to 3 times inviting them to participate in the study. The majority of the SA and AC groups in the UK reside in inner cities such as Birmingham and thus we anticipate the population to be representative of these ethnic groups. We aimed to recruit 3000 SA and 2000 AC patients. The sample size was determined based on the number of participants required to determine the prevalence of heart failure with reasonable precision (the principal aim of the E-ECHOES study). Verbal and written consent was obtained from all participants. Health-related quality of life (HRQL) was assessed using the EuroQoL EQ-5D questionnaire [6].

### EQ-5D

The EQ-5D is a validated, generic preference-based measure of health status that comprises a 5-question multi-attribute questionnaire [6]. Respondents were asked by a trained multi-lingual interviewer to rate severity of their current problems (level 1 = no problems, level 2 = some/moderate problems, level 3 = severe/extreme problems) for five dimensions of health: mobility, self-care, usual activities, pain/discomfort, and anxiety/depression. EQ-5D health states were converted into an EQ-5D score ranging from -0.594 to 1.0 (where 1 is full health and 0 is dead) using a set of weighted preferences produced from the UK population [13].

### Statistical analysis

Analyses were performed using SAS V9.2 (SAS Institute, Cary NC). A limited number of candidate explanatory variables were pre-specified: age, gender, ethnicity, smoking status (ever smoked versus non-smoker), alcohol consumption (occasional/regular consumption compared to non-drinker), exercise status (how often the participant engaged in any regular activity long enough to work up a sweat, defined as often, sometimes, rarely/never), body mass index (BMI), Index of Multiple Deprivation 2007; and history of: hypertension, arrhythmia, angina, myocardial ischemia, heart failure, diabetes, peripheral artery disease, stroke/transient ischemic attack (TIA), chronic obstructive pulmonary disease (COPD), asthma, cancer, arthritis, depression, number of comorbidities, marital status, religion and place of birth [1].

The relationship between EQ-5D score (response variable) and the candidate explanatory variables was assessed using a backward stepwise selection process with α = 0.05 as criteria for model inclusion in SAS statistical software (proc glmselect). Non-linear functional forms were considered for continuous candidate variables (log transformation, and, if that demonstrated a significantly better fit, a restricted cubic spline). The number of comorbidities was included as Log_e _(value + 1) term. More complex functional forms were included in the final model only when they provided a statistically significantly improved model fit assessed using Akaike's Information Criterion [14]. The final model included the selected variables in a mixed linear model (using proc mixed) with identity link, normal error and with general practice surgery as random effects [15]. A random effects approach was pre-specified for the final analysis to account for any practice related clustering of patient characteristics not otherwise captured by the model development process [16]. The mixed model approach extends the conventional analysis by enabling the effects of practice membership to be accounted for. The proportion of residual variance explained by the model was assessed.

The health status of SA and AC participants was compared with age-matched data from the UK population [9,10].

## Results

The baseline characteristics of patients enrolled in the E-ECHOES study are shown in Table 1. 13,097 subjects were screened, 10,902 were eligible and invited to participate and 420 (3.2%) did not meet the study inclusion criteria. 6,506 booked an appointment of which 5,408 (49.6%) completed the screening process and 5,354 (49.1%) had complete health status data (3442 South Asian and 1912 African-Caribbean participants). The mean age of participants was 60.8 years (SD 11.1 years), 2544 (47.5%) were male, 2676 (50.0%) had hypertension, 2158 (40.3%) had arthritis, 1563(29.2%) had diabetes, 415 (7.8%) had angina and 307 (5.7%) had a history of myocardial infarction.

**Table 1:** Patient Demographics

	South Asian	South Asian where EQ-5D score = 1	African-Caribbean	African-Caribbean where EQ-5D score = 1
n	3442	2402	1912	1447
Age (mean [SD], years)	59.7 (10.4)	58.2 (9.7)	62.7 (12.0)	61.1 (11.6)
Female, n (%)	1752 (50.90%)	1103 (45.92)	1058 (55.33%)	766 (52.94)
Systolic blood pressure (Mean, SD, mm Hg)	139.51 (19.85)	139.39 (19.84)	144.32 (19.88)	144.01 (19.50)
Diastolic blood pressure (Mean, SD, mm Hg)	80.87 (10.91)	81.46 (10.83)	81.99 (10.79)	82.54 (10.61)
Ever smoked, n (%)	801 (23.27)	572 (23.81)	826 (43.20)	624 (43.12)
Consumes alcohol (occasionally or regularly), n (%)	552 (16.04)	456 (18.98)	1306 (68.31)	1015 (70.15)
BMI Median (IQR)	27.56 (24.89 to 31.13)	27.27 (24.78 to 30.52)	28.99 (25.89 to 33.05)	28.56 (25.57 to 32.36)
				
				
Index of Multiple Deprivation 2007 Median, IQR [9]	54.74(39.02 to 61.39)	54.85 (39.29 to 61.50)	57.93(46.25 to 61.34)	57.93 (46.25 to 61.34)
				
				
Born in the UK, n (%)	55 (1.60)	47 (1.96)	309 (16.16)	266 (18.38)
Interviewed in English, n (%)	1508 (43.81)	1129 (47.0)	1904 (99.58)	1443 (99.72)
**Medical History, n (%)**				
Angina	328 (9.53)	194 (8.08)	87 (4.55)	47 (3.25)
Arrhythmias	58 (1.69)	31 (1.29)	46 (2.41)	18 (1.24)
Arthritis	1345 (39.08)	717 (29.85)	813 (42.52)	516 (36.57)
Asthma	414 (12.03)	254 (10.57)	199 (10.41)	136 (9.40)
Cancer	64 (1.86)	39 (1.62)	86 (4.50)	51 (3.52)
COPD	35 (1.02)	18 (0.75)	29 (1.52)	17 (1.17)
Depression	277 (8.05)	64 (2.66)	89 (4.65)	30 (2.07)
Diabetes	1060 (30.80)	693 (28.85)	503 (26.31)	337 (23.29)
Heart Failure	46 (1.34)	16 (0.67)	29 (1.52)	12 (0.83)
Hypertension	1570 (45.61)	987 (41.09)	1106 (57.85)	776 (53.63)
Myocardial ischemia	299 (8.69)	188 (7.83)	64 (3.35)	35 (2.42)
Peripheral artery disease	18 (0.52)	6 (0.25)	26 (1.36)	11 (0.76)
Stroke/TIA	157 (4.56)	65 (2.71)	67 (3.50)	24 (1.66)
Number of medical conditions (Median, IQR)	1 (1 to 3)	1 (0 to 2)	2 (1 to 2)	1 (1 to 2)
**Medication Use, n (%)**				
ACE Inhibitors	818 (23.77)	524 (21.82)	465(24.32)	316 (21.84)
Diuretics	639 (18.56)	384 (15.99)	616 (32.22)	418 (28.89)
Beta-blockers	470 (13.65)	294 (12.24)	236 (12.4)	150 (10.37)
Calcium Antagonists	613 (17.81)	364 (15.15)	716 (37.45)	498 (34.42)
Aspirin	1054 (30.62)	691 (28.77)	563 (29.45)	380 (26.26)
Warfarin	35 (1.02)	19 (0.79)	34 (1.78)	15 (1.04)
Digoxin	13 (0.38)	4 (0.17)	13 (0.68)	3 (0.21)
Lipid regulating drugs	1483 (43.09)	969 (40.34)	731 (38.23)	520 (35.94)

### Health-Related Quality of Life (HRQL)

The EQ-5D descriptive system was complete for all study participants. The mean EQ-5D score in South Asian participants was 0.91 (SD 0.18), median score 1 (IQR 0.848 to 1) and in African-Caribbean participants the mean score was 0.92 (SD 0.18), median 1 (IQR 1 to 1). Of the 5354 patients that participated in the E-ECHOES study 3849 (71.89%) reported no problems in any dimension. The characteristics of patients reporting full health status are shown in Table 1. The number of participants reporting problems in each dimension is shown in Table 2.

**Table 2:** Numbers (percentages) of South Asian (SA) and African-Caribbean (AC) respondents, reporting any problem (moderate or severe) for each EQ-5D dimensions by age group

	Number (%) of respondent reporting any problem (by age group)				
Age group, years	40-49	50-59	60-69	70-79	≥ 80
**EQ-5D dimension**					
**Mobility**					
SA	16 (2.34)	90 (6.62)	104 (14.59)	129 (23.63)	56 (40.0)
AC	14 (3.21)	19 (4.58)	44 (10.95)	92 (17.46)	50 (37.88)
**Self Care**					
SA	11 (1.61)	50 (3.68)	66 (9.26)	97 (17.77)	46 (32.86)
AC	11 (2.52)	15 (3.61)	35 (8.71)	76 (14.42)	41 (31.06)
**Usual activity**					
SA	22 (3.22)	87 (6.40)	102 (14.31)	150 (27.47)	54 (38.57)
AC	12 (2.75)	20 (4.82)	52 (12.94)	103 (19.54)	54 (40.91)
**Pain/discomfort**					
SA	76 (11.11)	226 (16.63)	160 (22.44)	153 (28.02)	43 (30.71)
AC	26 (5.96)	40 (9.64)	61 (15.17)	117 (22.20)	42 (31.82)
**Anxiety/depression**					
SA	85 (12.43)	162 (11.92)	71 (9.96)	48 (8.79)	10 (7.14)
AC	30 (6.88)	38 (9.16)	36 (8.96)	52 (9.87)	12 (9.09)

### Comparison with UK normative data

**Figure 1:**
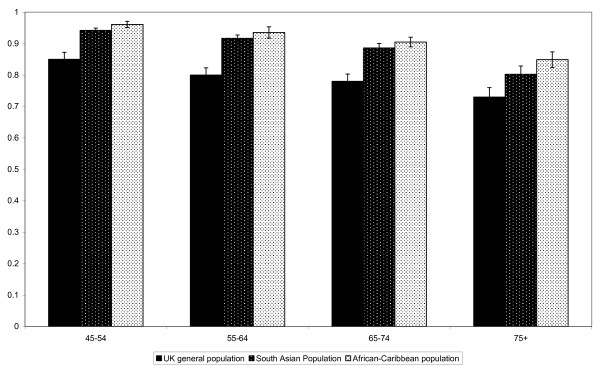
**Mean EQ-5D scores from a sample of the UK general population, assessed in 1993, by age**, [8]**compared to mean scores from South Asian and African-Caribbean participants in the E-ECHOES study (95% CI are indicated)**.

Compared with normative data from the UK general population substantially fewer African-Caribbean and South Asian participants reported problems with mobility, usual activities, pain and anxiety when stratified by age resulting in higher average health status estimates than those from the UK population (Figure 1) [9]. Those aged 60 and over did however report increased problems in self-care when compared with age matched UK population data [9].

### Patient Characteristics associated with HRQL

The results of multivariable analysis are shown in Table 3. The final model accounts for 19% of the residual variance. On average HRQL decreased with age, was lower in female participants and was reduced with increasing body mass index.

**Table 3:** Multivariable model results showing the change in EQ-5D score associated with each explanatory variable

Effect	Estimate	Lower 95% CI	Upper 95% CI	P Value
**Intercept**	0.267	0.097	0.436	0.004
**Age**	-0.003	-0.003	-0.002	< .0001
**Female**	-0.021	-0.030	-0.012	< .0001
**BMI**	-0.002	-0.003	-0.001	< .0001
**No medical history of:**				
Angina	0.039	0.018	0.059	0.0002
Arrythmia	0.075	0.042	0.107	< .0001
Arthritis	0.091	0.076	0.107	< .0001
Asthma	0.049	0.032	0.067	< .0001
Cancer	0.046	0.017	0.074	0.0016
COPD	0.060	0.019	0.101	0.004
Depression	0.166	0.146	0.187	< .0001
Diabetes	0.029	0.015	0.044	0.0001
Heart Failure	0.110	0.072	0.147	< .0001
Hypertension	0.039	0.021	0.056	< .0001
Peripheral Artery Disease	0.094	0.046	0.142	0.0001
Stroke or TIA	0.135	0.112	0.158	< .0001
Number of medical conditions*	0.072	0.043	0.101	< .0001
**Exercise **^#^				< .0001
Often	0.009	-0.009	0.026	0.324
Sometimes	0			
Rarely/Never	-0.031	-0.044	-0.018	< .0001
**Place of Birth^~^:**				0.0093
Africa	0.004	-0.024	0.032	0.77
Bangladesh	0.005	-0.021	0.031	0.69
Europe	-0.011	-0.038	0.016	0.43
India	0.001	-0.022	0.025	0.90
Jamaica	0.012	-0.010	0.034	0.29
Other	0.048	-0.081	0.177	0.47
Other Asia	0.055	-0.022	0.131	0.16
Pakistan	-0.016	-0.039	0.008	0.19
West Indies	0			

*Log_e _(number of conditions +1) ^#^p value for overall effect from F test < .0001; ^~^p value for overall effect = 0.0093

A medical history for a range of conditions was also associated with significantly reduced HRQL, most notably depression, stroke/TIA, heart failure, peripheral artery disease and arthritis. Participants who reported participating in regular exercise had improved HRQL. Place of birth was also associated with HRQL in the final model (Table 3).

## Discussion

This is the first large, community based study assessing the HRQL of South Asian and African-Caribbean subjects in the UK. Compared with normative data from the UK general population collected in 1993, substantially fewer African-Caribbean and South Asian participants reported problems with mobility, usual activities, pain and anxiety when stratified by age resulting in higher average health status estimates than those from the UK population (Figure 1). Our results suggest that the existing UK population 'norms' may be potentially outdated, and unrepresentative of current population health status, or inappropriate for use in these groups.

The observed differences may reflect changes in health status over time; however a contemporary sample of UK health status population data was unavailable. The results may also reflect difference in study design and implementation (Table 4), although comparisons between the E-ECHOES and UK population data were age-matched. On average participants in the E-ECHOES study have increased deprivation compared to the UK general population. We had anticipated that deprivation would be significantly associated with HRQL; however this was not observed based on these data. This may reflect homogeneity in deprivation scores observed within inner city Birmingham. One could argue that our findings reflect selection bias of healthy individuals however, all patients meeting the inclusion criteria were approached for the study and we achieved a relatively high response rate, with the sample being representative of that residing within the health authority area [17]. Analyses of those with health status data compared to those invited to participate did show that non-responders were likely to be younger males [18]. Since HRQL is reduced in females compared to males and deteriorates with age this seems an unlikely source of bias since it should have led to worse, not improved, estimates of population HRQL.

**Table 4:** Study characteristics of E-ECHOES compared to UK population norms [9,10]

	E-ECHOES	**UK Normative data **[8,9]
Study period	September 2006 to July 2009	Last quarter 1993

Population	SA and AC subjects from 20 practices in Birmingham, UK	Addresses in the UK to provide a sample representative of the general population with respect to age, gender and social class.

Sampling frame (n)	10902	6080 addresses

Number of participants (response rate, %)	5354 (49.1%)	3395 (55.8%)

Inclusion criteria	SA or AC aged > 45 years.	Age ≥ 18 years

Exclusion criteria	Dementia or terminal illness	Individuals in institutions, hostels, homes for the elderly or bed and breakfast

EQ-5D data collection	Interview	Questionnaire (as part of an interview process)

The results are also surprising given the increased risk of cardiovascular disease in these populations [19]. The prevalence of cardiovascular disease and diabetes appears to be higher in this cohort compared to an earlier study in the Midlands with a predominantly White British population, however the proportion of patients prescribed medications such as ACE-inhibitors and beta-blockers has also increased [20,21]. The management of chronic disease has improved in the UK during the time since the normative EQ-5D data was collected thus we may anticipate improvements in HRQL as a result [22,23].

One may question if our findings are a true reflection of health status in these communities. The HRQL in ethnic minority groups may be shaped by migration experiences, expectations, achievements and coping mechanisms, such as faith, prayer, and social support [24,25].

A challenge to our findings is that EQ-5D is self-completed in many settings in a paper format, including the collection of UK normative data [9,10,26]. In this study participants were asked by the researcher to respond to each question, as part of the study interview, which was recorded directly onto the study database. This allowed interpretation if necessary and enabled us to obtain a 100% response rate for the questionnaire. However, evidence suggests that participants are more likely to yield higher HRQL scores in an interview situation compared to paper based self administration [27].

Our model demonstrates that HRQL is significantly reduced with ageing, in females, in those with increased BMI and is reduced in the presence of a range of medical conditions. Compared with normative data from the UK general population African-Caribbean's and South Asian's reported fewer problems in each of the dimensions of the EQ-5D, except self-care where more problems were reported in the elderly [9,10]. Our model results demonstrate that depression leads to significant reductions in HRQL (16%), however the number of participants reporting a prior medical history of depression was lower than anticipated (8.1% SA; 4.7% AC) and may reflect the stigma of mental illness. Arthritis is associated with significant reduction in HRQL. This condition was highly prevalent in both populations and should be considered as a target for improved management strategies.

A limitation of the model is that 'main language spoken' was not pre-specified as a variable in our analysis plan, since we did not anticipate that this would have a significant relationship with health status. However, the questions, particularly those relating to self-care, usual activities and depression may not have common cultural or linguistic interpretation, thus the underlying constructs may not be equivalent [28,29]. The consistency of delivery, in particular wording of questions and item presentation, may be questioned due to the range of researchers being involved and the number of languages necessitated in the study. We cannot rule out that respondents misunderstood or chose not to disclose their true health status due to social desirability bias [30,31]. However, the 'best' health status scores were observed in the African-Caribbean group, where the interview took place in English for 1904 (99.58%) of participants.

A further limitation is that we used UK general population weights to generate the EQ-5D scores [13]. There is evidence to suggest that different populations' value health states differently but, currently no valuation has been undertaken in these minority groups in the UK [32].

Despite these potential limitations this is the first study to report the health status of these minority ethnic groups based on a large community sample in the UK. Our findings bring into question the appropriateness of the widely used UK population based health status survey results from 1993 in these populations and more generally the use of these potentially outdated data to inform health policy in the UK [9,10]. There is limited evidence on the validity and reliability of the EQ-5D in these populations [29]. Further research is necessary to assess this and to explore the effect of mode of administration on response in these groups, and to assess the potential impact on results of economic evaluation [33].

## Conclusions

The reported HRQL of these minority ethnic groups was substantially higher than anticipated compared to UK normative data. Participants with chronic disease, notably those with arthritis, depression, heart failure or stroke, experienced significant reductions in HRQL and should be a target for health intervention.

## Competing interests

NF, GL, and MC have received funding for research, consulting and speaking from a range of companies which manufacture treatments for heart failure or other cardiovascular therapies.

## Authors' contributions

All authors contributed to the study design. MC led the statistical analysis and drafted the manuscript. All authors contributed to the final draft and approved the final version for submission.

## Pre-publication history

The pre-publication history for this paper can be accessed here:

http://www.biomedcentral.com/1472-6963/12/101/prepub
